# Management of Opioid Addiction With Opioid Substitution Treatments: Beyond Methadone and Buprenorphine

**DOI:** 10.3389/fpsyt.2018.00742

**Published:** 2019-01-18

**Authors:** Florence Noble, Nicolas Marie

**Affiliations:** ^1^CNRS ERL 3649, “Neuroplasticité et thérapies des addictions”, Paris, France; ^2^INSERM UMR-S 1124, Paris, France; ^3^Centre Universitaire des Saints Pères, Université Paris Descartes, Paris, France

**Keywords:** addiction, morphine, buprenorphine, methadone, substitution treatment

## Abstract

With the opioid crisis in North America, opioid addiction has come in the spotlight and reveals the weakness of the current treatments. Two main opioid substitution therapies (OST) exist: buprenorphine and methadone. These two molecules are mu opioid receptor agonists but with different pharmacodynamic and pharmacokinetic properties. In this review, we will go through these properties and see how they could explain why these medications are recognized for their efficacy in treating opioid addiction but also if they could account for the side effects especially for a long-term use. From this critical analysis, we will try to delineate some guidelines for the design of future OST.

## Introduction

When people talk about opioid problems or addiction to opioids, they think of opioids that some people get on the street, such as heroin, with the idea that only a minority of persons is concerned. But the truth is very different and anyone who uses an opioid can develop addictive behaviors. This is not a specific problem for heroin users as opioids are very useful molecules and powerful medications that are generally prescribed to relieve severe pain. Thus, problematic opioid use may also include the misuse of prescription opioid medications, such as oxycodone, morphine, or codeine, or the use of a drug for which no personal prescription has been received. As a result, the number of people over-using or dependent on opioids is increasing dramatically and is a public health problem. Over the past few years, both the U.S. and Canada have seen a spectacular increase in opioid overdose rates. From 1999 to 2016, more than 350,000 people died from opioid overdose in U.S. (https://www.cdc.gov/drugoverdose/epidemic/index.html). This so-called “opioid epidemic” or “opioid crisis” started in the 1990's with the conjunction of different factors including propaganda by pharmaceutical companies claiming that their opioids had a low liability to induce addiction mainly because of the extended release formula, and a better pain management which led to a widespread use of opioid drugs for the treatment of moderate pain (1, 2).

Behind these perfectly quantified data, there is another figure that is difficult to quantify, but which is most certainly very high, people with opioid addiction. This crisis shed light on the weakness of available treatments to manage opioid addiction. Two main medications—the opioid substitution treatments (OST) are used: buprenorphine and methadone. After a rapid review of neurobiology of opoid addiction, we will review some properties of these OST that could explain why they have a certain success [for an extensive review on methadone and/or buprenorphine, see (3)]. However, this success is only relative (relapses very often occur even when patients are under OST) and as banning opioids is not an option, it is therefore important to discuss the future of opioid research. A table with the opioids cited in the present review is included to facilitate the reading of the manuscript (Table 1).

**Table 1:** Examples of preclinical and clinical opioid drugs.

**Ligand**	**Selectivity**	**Activity**
Morphine	MOPr >> KOPr	Agonist	Analgesia Respiratory depression
Heroin	*MOPr >> KOPr*	*Agonist*	Fast penetration in the brain *Acts through its mainly active metabolite, morphine*
Buprenorphine	MOPr DOPr KOPr	Partial agonist AntagonistAntagonist	Reduces withdrawal Low risk of respiratory depression
Methadone	MOPr	Agonist	Reduces withdrawal, risk of respiratory depression
TRV130	MOPr	Agonist (biased toward G protein)	
PMZ21	MOPr > KOPr > DOPr	Biased agonist Antagonist Weak agonist	Analgesia
Cebranopadol	MOPr, KOPr, NOPr > DOPr	Agonist (partial at KOPr)	Reduced respiratory depression
AT-121	MOPr, NOPr > DOPr KOPr	Partial agonist Partial agonist	
Naloxone	MOPr, DOPr, KOPr	Antagonist	Blocks euphoric effects Reverses respiratory depression
Naltrexone	MOPr, KOPr > KOPr	Antagonist	
PL37, PL265	MOPr, DOPr (via enkephalins)	Inhibit enkephalins degradation	Analgesia Lack opioid-associated side effects

## The Neurobiological Basis of Opioid Addiction

It has been known for a long time that opioids such as morphine, heroin, and derivatives induce numerous pharmacological responses, including analgesia, dependence, respiratory depression or euphoria (4, 5). From these observations, evidence that different opioid drug effects could only be explained by the existence of stereospecific receptors has emerged. In the 1970s, the endogenous opioid receptors were discovered (6–8), followed by the characterization of the endogenous opioid peptides (9). Since these identifications numerous studies have been conducted in the opioid field.

Historically, three opioid receptors have been characterized, mu (MOPr), delta (DOPr), and kappa (KOPr). Additional receptor types have been identified, but are no longer considered as “classical” opioid receptors (e.g., sigma, nociceptin/orphanin receptor, NOPr) (10). The three opioid receptors were cloned in the early nineties (11–14). Since this period, several knockout mice lines, each harboring deletions of the genes encoding a particular opioid receptor, have been used to clarify the specific role of the different receptors *in vivo* and in many physiopathological conditions (15). In this review the focus will be on reward and addiction.

It is well-known that all drugs of abuse increase extracellular dopamine levels in the nucleus accumbens (Nac), either directly (e.g., cocaine and amphetamine directly target dopamine transporters), or indirectly (e.g., opioids decrease GABA release in the ventral tegmental area, leading to an activation of dopamine neurons). Several lines of evidence indicate that MOPr play a key role in mediating the rewarding effects of opioids, while the role of DOPr remains debatable, and KOPr are considered to have opposite functions to those of MOPr in the regulation of reward and addiction. KOPr agonists have dysphoric and aversive effects in humans and rodents (16, 17), in good agreement with decreases in dopamine release in the Nac observed following injection of selective agonists in this brain structure (18).

The pharmacological responses induced by opioids (e.g., conditioned place preference, intravenous self-administration, locomotor activity, analgesia) are abolished in MOPr knockout mice, demonstrating that MOPr represent the primary *in vivo* molecular target for these ligands (15). Morphine-induced conditioned place preference in an unbiased procedure is also reduced in DOPr knockout mice (19, 20), but these animals show normal motivation to obtain morphine in intravenous self-administration paradigm (20). These results, combined with other data obtained from other experimental approaches suggest that morphine reward and motivation to obtain opioids are intact in DOPr knockout mice, however drug-context association is more certainly impaired.

Both with most clinically useful (e.g., morphine, fentanyl, oxycodone) and most largely abused (heroin) opioids, opioid-use disorder is a public health problem. The number of opioid prescriptions sharply increased in the past two decades, increasing risks for addiction and overdoses. Addiction to prescribed opioids is associated with transition to illicit opioid use like heroin (21), and overdoses have strongly risen since the 1990s (22). As mentioned earlier the notion of “opioid crisis” or “opioid epidemic” has emerged in North America, and to a lesser extent in Australia (23). European countries appear to be less affected (24), but even if the risk in Europe appears relatively limited, vigilance is needed (25).

Opioid addiction is a brain disorder, involving alterations in neuronal circuits with complex neuroadaptative mechanisms that lead to dependence, craving, and relapse; thus contributing to the maintenance of drug use. Until now, no medication can reverse the drug-induced changes observed in the brain that are involved in the relapsing nature of opioid-use disorders, even after a protracted abstinence. Currently, the therapeutic approach using an agonist strategy with methadone and buprenorphine, has shown physical and psychosocial improvements in drug users, but these molecules possess MOPr agonist properties which limit their clinical usefulness, as described below.

## Characteristics of the Opioid Substitution Treatments

### The Way They Reach Their Target: Pharmacokinetic Properties

The therapeutic action of a compound strongly depends on its pharmacokinetic properties (26). The opioid users seek a rapid and intense euphoria which is obtained with heroin, which is a prodrug. Indeed, although it has a low affinity toward MOPr, its action is mainly mediated by its metabolites including morphine (27, 28). The intense and rapid euphoria following heroin administration is partly due to its high lipophilic nature, enabling the molecule to readily cross the blood-brain barrier (29). Another very important characteristic that determines the fast action of heroin is the route of administration: the intravenous route being the fastest (30). OST are both oral medications, methadone as a syrup or pills and buprenorphine as sublingual tablet or films. Methadone has a good oral bioavailability (between 40 and 95%) (31), conversely, buprenorphine has a poor oral bioavailability. In any case, both oral and sublingual routes allow the OST to diffuse slowly, thus avoiding peak effects which contribute to addiction. Therefore, after ingestion, the peak effects and peak plasma levels are reached between 1 and 6 h for methadone (average: at 4 h) (32), whereas the peak levels occur ~1 h after buprenorphine administration (33, 34). One of the mandatory features to be a good OST is that it needs to have slow metabolism and elimination profiles which avoid patients experiencing withdrawal. Methadone and buprenorphine fulfill these criteria, with an average half-life of 22 and 32 h, respectively (31, 33), therefore these medicines are taken once a day, which favors the observance. Opioid pharmacokinetics are influenced by their interaction with enzymes that metabolize xenobiotic, such as cytochromes P450 and efflux pumps. For instance, the two diphenylpropylamine opioids loperamide and methadone, which display similar structures, have different fates once administered. Whereas, methadone transport to the brain is partly restricted by the multidrug resistance protein 1 (MDR-1) (35), loperamide is unable to cross the brain blood-brain barrier due to the presence of the same efflux pump (36) showing that loperamide is a better substrate for MDR-1 than methadone. Many pharmacogenetic studies of cytochromes P450 such as CYP450 3A4 (one of the main cytochromes involved in OST metabolism) or efflux pumps have been conducted to explain the variability in OST dosing. Overall, it appears that although some variants of these genes are associated with OST plasma levels, their influence on dose requirement is very low (37). OST pharmacokinetics is more likely to be influenced by co-prescribed drugs, which interact with their metabolism. For instance, delavirdine, an antiretroviral medication used in HIV treatment, inhibits CYP450 3A4 and thus induces an elevation of methadone plasmatic concentration and drug delayed clearance (38).

### The Way They Interact With the Target: Pharmacodynamic Properties

Methadone and buprenorphine bind MOPr with a higher affinity as compared to morphine. Therefore, when a patient under OST uses heroin, its effects will be reduced, as the morphine concentration in the brain will not be high enough to displace methadone or buprenorphine from the receptor. This highlights the issue of the optimum dose of OST, so each patient must have a sufficiently high brain concentration to avoid withdrawal symptoms. In addition, buprenorphine has a very low receptor dissociation rates (39–41) conferring a long duration of action (which contributes to its long half-life) and reinforcing its inability to be displaced by other opioids. Opioid overdoses cause death by respiratory depression: indeed, whereas tolerance to analgesia develops rapidly, tolerance to respiratory depression is far weaker and slower to appear (42). Methadone is a full agonist at the MOPr (43) and its potency and efficacy increase the risk of overdose, thus requiring this drug to be administered to treat opioid dependency only in designated medical units with trained staff. Buprenorphine has a particular pharmacological profile and is described as a MOPr partial agonist (44). In pioneering studies conducted in rodents, buprenorphine displayed a ceiling effect, exerting only partial analgesia compared to morphine or more effective agonists (45). Nevertheless, more recent studies have not shown this ceiling effect in other species such as humans where buprenorphine is quite powerful (46)—probably because there is a greater MOPr reserve [i.e., more spare receptors (47)]. The ceiling effect is probably rather more specific to the target system (e.g., respiration) than to the species (48) and may be explained by differences in the receptor reserve in the different pathways (pain, respiration…), probably explaining the lack of severe respiratory depression at analgesic doses with this drug (46). As a consequence, it was allowed to prescribe buprenorphine as an ambulatory medicine in many countries including UK, France, USA. Buprenorphine is also depicted as a KOPr antagonist, which might contribute to its antidepressant effect (49).

## Why Searching for New Treatments for Opioid Addiction?

It is undeniable that the actual OST, methadone and buprenorphine, have brought a substantial benefit in the opioid addiction treatments. Indeed, when associated with a risk reduction policy they substantially reduced death by overdoses and the transmission of blood-borne diseases. They help addicts to follow their recovery program and contribute to their social reintegration. OST were also shown to preserve immune (50) and memory (51) functions, have positive effects on psychopathology (52, 53) and reduce polyabuse (54).

However, like any other medications, OST are not fully effective as many patients under OST might still relapse (55, 56), and because they are MOPr agonists they may be misused (57). The promised safety of buprenorphine was challenged as soon it arrived on the market and for example in France, several death cases were reported where buprenorphine was diverted (intravenous use). Whereas, several of these cases included the concomitant use of buprenorphine with other depressants of the respiratory system (ethanol and/or benzodiazepines), some of them reported only buprenorphine use (58). More recently, when gabapentin was used with opioids a substantial increase in the risk of opioid-related death was measured (59). Beyond the high risk of fatal respiratory depression (see above), methadone is associated with prolongation of the electrocardiographic QT interval (60, 61). However, the link to cardiac dysrhythmia and sudden cardiac death remains an open question. Indeed, recent studies did not confirm the role of methadone in sudden cardiac death (62) as it was previously suspected.

Many side effects have been reported with these OST such as a decrease of cognitive performance (63) or sexual dysfunction in men (64, 65). Finally, as they remain MOPr agonists, they will contribute to maintain—very likely to a lesser extent—the allostasis generated by previously abused opioids. In rodents, a short treatment (5 days) with buprenorphine or methadone is able to induce behavioral and neurochemical modifications until 35 days after withdrawal (66, 67). It therefore appears necessary to find new MOPr agonists, or new combinations of MOPr agonists and other ligands, that would not induce the neuroadaptations responsible for the harmful effects of opioids (e.g., addiction, respiratory depression), and would therefore gradually restore homeostasis, thus allowing for instance a complete escape from addiction.

On the other hand, to avoid buprenorphine diversion, different formulations of buprenorphine are currently evaluated and usually consist of transdermal patches, subcutaneous depot injections, or subdermal implants (68). An alternative strategy to limit diversion is to combine buprenorphine with an opioid antagonist, naloxone (suboxone). Naloxone has a poor oral bioavailability, but when injected intravenously (in the case of misuse), it will precipitate withdrawal. Human studies shown that it has a reduced abuse potential (69), however recent preclinical (70) and clinical (71, 72) data questioned the lower level of rewarding properties of intravenous suboxone.

## Some Leads on the Future of Opioid Research

The “opioid crisis” dramatically exposes the need for more research in at least two main directions. One is to find better opioid analgesics with less and even virtually no addictive potential. The other direction is the discovery of new medications to treat opioid addiction. We will discuss these two directions focusing on opioid-based drugs.

Since the 1990's, studies have demonstrated that different ligands could induce (or select) different receptor conformations that could promote different signaling pathways. This concept is now known as biased agonism or functional selectivity (73). For opioid receptors, this notion combined with the pioneer work of Bohn and co-workers paved the way to design new opioids. It is now well-established that following ligand binding, MOPr activation could result in the activation of multiple downstream pathways through either G protein dependent processes (e.g., regulation of ion channels, adenylate cyclase inhibition) or G protein independent processes (e.g., beta-arrestin signaling). Beta-arrestin is a protein that binds the activated and phosphorylated receptor and is responsible for its desensitization and endocytosis (74). Bohn and co-workers found that in beta-arrestin-2 knockout mice, morphine analgesia was increased and prolonged (75, 76) with a decrease of respiratory depression and acute constipation (77). Therefore, it has been suggested that biased opioid agonists toward G protein pathway will retain analgesic effects with a reduction of side effects including tolerance mediated by beta-arrestin activation. This last point is of particular importance as tolerance, by increasing the dose required to induce the same effects, will contribute to dependence and overdose. So, recently few opioid biased agonists for the treatment of pain have been developed including TRV130, a compound recently entered in phase 3 to treat moderate and severe acute pain (78). This molecule is biased toward G proteins and shows less tolerance and respiratory depression as compared to morphine (79). Using the recent discovery of MOPr structure (80), Manglik and co-workers discovered PMZ21 a molecule that displays a protracted analgesia as compared to morphine and like the TRV130 has no rewarding effects in the conditioned place preference paradigm (81). However, this lack of rewarding effects has been recently challenged by Altarifi and colleagues who found that TRV130 reduced the threshold of intracranial self-stimulation (82). These results are not surprising as these molecules selectively target MOPr, so alternative strategies are currently considered such as targeting multiple opioid receptors to reduce some side effects and increase efficacy (83). For instance, cebranopadol a mixed MOPr/DOPr/KOPr/NOPr receptor agonist was found to be efficient in acute and chronic pain and development of tolerance was delayed as compared to equianalgesic doses of morphine (84). More recently, Ding et al. reported the discovery of AT-121, a MOPr/NOPr mixed agonist with analgesic effects in non-human primates and a lack of common opioid-associated side effects such as physical dependence, abuse potential, respiratory depression, and opioid-induced hyperalgesia (85). Finally, instead of activating opioid receptors with synthetic compounds that could results in unwanted effects (due to overstimulation in many target systems) the use of endogenous ligands has been proposed through the blockade of the catabolism of the endogenous peptides. This approach was developed by Roques and co-workers in the 1980's who published the first study showing that blocking enzymatic degradation of enkephalins enhances their physiological effects (86). It has the advantage to target only the structures where enkephalins are expressed, thus explaining why multiple preclinical studies demonstrated that these compounds are as effective as morphine to produce analgesia but without promotion of tolerance, physical dependence, constipation or respiration depression (87). Indeed, enkephalins are highly expressed in pain-control centers (88) whereas they are found in low amount in respiratory centers (89) or locus ceruleus (90), a structure involved in the expression of opioid physical dependence (91). At the moment, two of these molecules, PL37 and PL265, are in clinical development for treating acute and chronic pain.

Regarding the treatment of opioid addiction, no real progress has been made since the introduction of methadone and buprenorphine and most of the current research consists of work related to these compounds or other marketed opioids such as modifying the formulation to obtain slow-release compounds. For instance, it has been proposed to use slow release morphine for patients who cannot tolerate methadone (92).

Recently, some opioid antagonists (e.g., naltrexone, naloxone) have been approved for opioid addiction but only for abstinent patients because of the risk of withdrawal. They have multiple benefits: lack of reinforcing effects, blockade of the euphoric effects of opioids, relative safety (no respiratory depression) (93). Even so, the adherence to these medications is generally poor, thus limiting their efficacies for the prevention of relapse in patients with opioid-use disorder. To circumvent this low treatment observance, an injectable extended-release naltrexone was developed. The first meta-analysis on its efficacy mainly revealed that, unsurprisingly, the success of extended-release naltrexone was higher in opioid detoxified patients. However, when randomization occurred after detoxification, extended-release naltrexone showed similar efficacy to buprenorphine, whereas when randomization occurred prior to detoxification, buprenorphine efficacy was superior (94). The fact remains that opioid antagonists are very efficient in emergency medicine, by preventing opioid overdose fatalities (95). Naloxone is actually the only opioid antagonist approved for treating opioid overdose. Its efficacy is based on a rapid onset of action via intravenous route (2–3 min) (96), but its shorter half-life than that of most opioid agonists, requires multiple injections or continuous administration to reverse respiratory depression. A recent study showed that it was also able to reverse buprenorphine-induced respiratory depression (97). It is noteworthy that fast opioid detoxification in opioid-dependent patients might lead to acute opioid withdrawal syndrome accompanied by catecholamine releases, responsible for cardiac and respiratory functions impairment (98).

## Conclusion

This review was focused on opioids, but knowing whether if they will remain the gold standard in pain management is an open question considering the opioid crisis. In addition, long-term treatment with OST, more than restoring the neurobiological equilibrium disturbed by the opioid misuse, will maintain drug-induced neuroplastic changes. So, besides the short and mid-term necessary research on the discovery of safer opioids, other pharmacological strategies have to be envisioned based either on different use of existing treatments or on other neurotransmitter systems with the objectives of having painkillers devoid of any activity on the reward system.

## Author Contributions

NM and FN wrote the manuscript. All authors take responsibility for final content. All authors read and approved the final manuscript.

### Conflict of Interest Statement

The authors declare that the research was conducted in the absence of any commercial or financial relationships that could be construed as a potential conflict of interest.
